# Should Parents Only Use One Language with Their Autistic Children? The Relations Between Multilingualism, Children‘s Social Skills, and Parent-Child Communication

**DOI:** 10.1007/s10803-024-06347-w

**Published:** 2024-05-29

**Authors:** Maïte Franco, Andreia P. Costa

**Affiliations:** Institute for Health and Behaviour, Department of Behavioural and Cognitive Sciences, Maison des Sciences Humaines, University of Luxembourg, 11 Porte des Sciences, Esch-sur-Alzette, L-4366 Luxembourg

**Keywords:** Autism, Multilingualism, Social Skills, Language use, Parent-child Communication

## Abstract

**Purpose:**

Parents of autistic children are often advised to use only one language to simplify their child’s language acquisition. Often this recommendation orients towards the geographically predominant language, which may cause difficulties especially for minority-language families. On the other hand, scientific evidence suggests that multilingualism does not hinder language acquisition and that communicating in exclusively foreign languages may even impede social interaction. Therefore, we investigated how parent language use is linked to the social skills of 68 autistic children and to their parents’ ability to feel comfortable, authentic, and free to express themselves.

**Methods:**

Data was collected online, using parent-report questionnaires from parents of 25 different nationalities in the European context, assessing children’s language, autistic traits (AQ-C), social skills (SRS-2), and parent-child communication.

**Results:**

Language use was not found to significantly relate to social skills in children. However, parents using their mother tongue, either only their mother tongue or in combination with other languages, reported feeling significantly more comfortable and more authentic than parents using exclusively foreign languages, either one or many. There were no significant differences between monolingual and multilingual families regarding parents’ feelings in regard to their language use.

**Conclusions:**

Our findings may encourage specialists to consider multilingualism more often and consult with parents whether monolingualism is worth risking the negative outcomes we have found. Especially, since advising parents to raise their child multilingually may facilitate access to therapeutic treatment, childcare, and social interaction in multilingual societies and families and subsequently improve support and orientation for stakeholders.

**Supplementary Information:**

The online version contains supplementary material available at 10.1007/s10803-024-06347-w.

A person’s socialization (i.e., the development of their identity, ethical values, behavioural patterns, and motivation) is directed by their social environment, such as people and institutions (Zimbardo & Gerrig, 2008) and intrapersonal factors, such as temperament and biological features (Thomas & Chess, 1977). For a positive socialization, children must learn appropriate social skills through their parents, peers, and personal role models (Ladd, 2005). Those include expression of empathy, negotiation skills, problem solving skills, generosity, and helpfulness (Lynch & Simpson, 2010). The acquired social skills serve as guidelines for subsequent academic and professional success (Deming, 2017; Meier et al., 2006), social life, and psychological as well as physical well-being (Beauchamp & Anderson, 2010; Cacioppo, 2002).

However, autistic people^1^ are at risk of poor socialization, as autism is defined by persistent difficulties in social communication and social interaction across different contexts (American Psychiatric Association [APA], 2013). Autistic children have a higher risk for exclusion than their neurotypical (Chamberlain et al., 2007; Humphrey & Lewis, 2008; Maïano et al., 2016; Schroeder et al., 2014) and otherwise neurodiverse peers (Humphrey & Hebron, 2015; Sreckovic et al., 2014), and have very limited relationships in adulthood (Howlin et al., 2004). Difficulties with socialization represent an important impairment for autistic people as they might lead, for example, to academic and professional underachievement (Howlin & Goode, 1998) and mood and anxiety problems (Myles, 2003; Tantam, 2003), regardless of cognitive and language abilities (Carter et al., 2005). Their difficulties may include struggles with the use and interpretation of figurative language and nonverbal communication, including eye-to-eye gaze (Neumann et al., 2006), facial expressions, body posture, and gestures. Furthermore, people on the spectrum may experience difficulties with the ability to adopt the conversation partner’s perspective (MacKay et al., 2007), speech prosody (Peppé et al., 2007), and social pragmatics (Philofsky et al., 2007), such as turn-taking in conversations (Chiang et al., 2008). Growing up, autistic people may develop even more distress, as social interactions tend to become more complex and they might acquire greater awareness of their difficulties (Schopler & Mesibov, 1983; Tantam, 2003). Accordingly, autistic adolescents report more loneliness, less social support, and express more desire for greater peer social interaction than neurotypicals (Bauminger & Kasari, 2000).

## Multilingualism and Social Skills

The acquisition of social skills and resulting socialization may be influenced by language use (Brinton & Fujiki, 1993; Damico & Damico, 1993). According to *The socialization paradigm* by Ochs and Schieffelin (1984), a person acquires socio-cultural knowledge (i.e. knowledge about social values and normative behavior of a given society) via language and develops linguistic knowledge via the process of socialization. This implies that language acquisition and socialization are bilaterally related. Accordingly, social problems were among the first to be noted as negative outcomes of communication disorders in the early speech-language pathology literature (Koepp-Baker, 1937; Travis, 1936). After all, we use language to make interpersonal contact and navigate our social interactions (Gallagher, 1991).

Especially multilingualism, the ability to communicate in two or more languages or dialects (Horner & Weber, 2017), has gained attention in research regarding the link between language and social skills. In addition to reports of cognitive and executive benefits in relation with multilingualism (Bialystok, 2009; Carlson & Meltzoff, 2008; Crivello et al., 2016; Filippi et al., 2015; Kovács & Mehler, 2009a, b), a few studies find additional benefits to theory of mind abilities and social aspects of communication skills (Goetz, 2003; Liberman et al., 2017). For example, children who have been exposed to several languages, even when monolingual themselves, were found to have an advantage in understanding the intended meaning of their conversation partners (Fan et al., 2015; Liberman et al., 2017). When being exposed to several languages, children are required to acknowledge their conversation partners’ mindset and language abilities, by taking their perspective (Liberman et al., 2017). Thus, multilingual people may have greater metalinguistic understanding, linguistic control, and socio-linguistic awareness, resulting in better representational abilities and understanding of others knowledge (Goetz, 2003).

## Multilingualism and Autism

Due to overall globalization, immigration, and multicultural influences, multilingualism has become more of a prerequisite rather than an option (De Houwer, 2009). Over 50% of the world population is estimated to speak at least two languages (Grosjean, 2013; Kremer-Sadlik, 2005) and many countries like Belgium, Canada, Luxembourg, Singapore, and Switzerland have multiple official languages. However, parents of autistic children are often advised to use only one language when communicating with their child (Harlin & Paneque, 2006; Kremer-Sadlik, 2005; Wharton et al., 2000; Yu, 2013, 2016).

### Mono- vs. Multilingualism

Specialists dissuading multilingualism may base their concerns on the *developmental interdependence hypothesis* and the *threshold hypothesis* by Cummins (1979). The *developmental interdependence hypothesis* states that a child’s skills in their second language are related to their skills in their first language. The *threshold hypothesis* claims that to benefit from bilingualism and avoid cognitive disadvantages, a certain threshold of proficiency in the first language must be overcome before acquiring a second one. Thus, if the acquisition of the first language is delayed, children are not likely to successfully acquire a second language and the risk to develop cognitive difficulties increases. Autistic people tend to struggle with language acquisition and approximately 25–50% are non- or minimally verbal (Rose et al., 2016; Baghdadli et al., 2012). They often have difficulties with linguistic pragmatics and show difficulties in understanding or using figurative speech, including metaphors, irony, or sarcasm (Kerbel & Grunwell, 1998; Philofsky et al., 2007; Shaked & Yirmiya, 2003; Tager-Flusberg, 2003). Their skills in linguistic abilities such as syntax, semantics, phonology, morphology, and vocabulary, however, are often as expected for their age if the person is verbal (Baker, 2013).

However, in opposition to the theories by Cummins (1979), the *translanguaging theory* (García & Wei, 2014) conceptualizes multilingualism as dynamic. It states that people have only one overall semiotic linguistic repertoire for all languages. This repertoire is said to consist of various lexical, grammatical, and morphological linguistic features, that are selected and deployed in a language according to the (social) context (Otheguy et al., 2015). Thinking of multilingualism as several distinct cognitive representations of languages would thus not match people’s daily experience (Yu, 2016). Multilingualism would rather be an integrated construct, which cannot be separated into discrete parts. Advising parents to monolingualism would thus impose them an unreachable goal.

Accordingly, several studies found that multilingual and monolingual children on the spectrum do not show significant differences on autistic traits, a broad variety of language skills, social, behavioural, and communication outcomes (Dai et al., 2018; Gonzalez-Barrero & Nadig, 2018; Siyambalapitiya et al., 2022; Uljarević et al., 2016). In a review on the comparison between multilingual and monolingual autistic participants, multilingualism was reported to have positive effects on communication and social functioning in autistic participants (Uljarević et al., 2016). Li and colleagues (2017) found that multilingual children on the spectrum performed as well as their monolingual autistic peers regarding executive functions and social and communication skills. Bilingual toddlers on the spectrum were even found to vocalize, use gestures, and show aspects of pretend play significantly more often than their monolingual autistic peers (Valicenti-McDermott et al., 2012).

## Autism, Multilingualism, and Social Skills

### Reasons for Mono- or Multilingualism

Parents who report concerns about multilingualism and who choose a monolingual upbringing report as decisive factors pronounced autistic traits, the advice they received from practitioners, and the fear of losing the support and access to services altogether. They worry about confusing and not being able to support the child and losing the support of their family and friends. They consider communication with extended family and prioritizing the culturally dominant language as important factors (Howard et al., 2021; Kay-Raining Bird et al., 2012). Additionally, they report to feel torn, with feelings of guilt when not meeting the professional recommendation and mixing up languages instead of using only one language (Jegatheesan, 2011).

On the other hand, parents with positive attitudes towards multilingualism, report as main reasons the multilingual living context and thus communication with other people, beliefs about cognitive and social awareness benefits, and more opportunities in children’s future life and professional career (Howard et al., 2021; Kay-Raining Bird et al., 2012). Interestingly, while some preferred raising their children monolingually due to the presence of impaired language and cognitive skills, others raised their children multilingually, despite notable language learning difficulties. They believe that raising their children multilingually supports the acquisition and expansion of linguistic skills (Jegatheesan, 2011; Kay-Raining Bird et al., 2012).

Reports from autistic self-advocates, contribute to this line of findings. In an online survey including 297 autistic adults, multilingual participants reported their social life as more satisfactory than the monolingual participants (Digard et al., 2020). Autistic people reported that their multilingualism enabled relations with family and friends, interaction with the international autistic community, employment, and educational opportunities, as well as positive psychological outcomes, such as increased confidence and joy (Nolte et al., 2021).

### The Importance of the Mother Tongue

If multilingualism is dissuaded, professionals often advise parents to use the culturally predominant language to enhance access to interventions and their efficacy (Yu, 2016). For example, in the US, physicians, speech therapists, teachers, and other professionals tend to advise immigrant families with autistic children to use only English with their children, often regardless of the parents’ English proficiency (Kremer-Sadlik, 2005; Solomon, 2008; Wharton et al., 2000; Yu, 2009). In a study including 22 Hispanic immigrant mothers, 13 families had been advised to opt for English only, even though 11 of them had at least one primary caregiver, who was not able to communicate in English (Ijalba, 2016). Imposing monolingualism in a multilingual family may be detrimental for the family members’ wellbeing, communication, and social interaction within the family (Baker, 2013; Ijalba, 2016; Jegatheesan, 2011; Kremer-Sadlik, 2005). Thus, this type of recommendation leads to mixed opinions in parents regarding their choice of language use.

Parents including their mother tongue, stressed the crucial importance of it for their children’s cultural identity (Howard et al., 2021). If their children would not acquire the family heritage language(s), their integration and participation in cultural activities of their heritage community would be notably hindered (Jegatheesan, 2011). Some parents believed that the heritage language would help their children acquire cultural values, such as family solidarity, respect, duty, and the importance of their relatives. Immigrant parents from Cuba, Russia, Denmark, and France who were told to use English for communicating with their child on the spectrum were found to be less affectionate and did not encourage their children’s speech as much as they did when using their heritage languages (Wharton et al., 2000). When heritage language was used, interactions between parents and their children were found to be very affectionate and filled with humour and, overall, more engaging in comparison to when English was used. Limited English proficiency and neglect of parents’ heritage language impaired the dynamic in minority-culture families. Due to tongue-tied verbal exchange and impeded social interactions, some of the children were left out of family conversations held in the family heritage language(s) (Kremer-Sadlik, 2005; Jegatheesan, 2011; Yu, 2009). Families who are not fluent in the recommended language may experience frustration, miscommunication, and restrictions in their expressions (Yu, 2016). Words of affection, small comments, and instructions may not be properly translated, thus creating a different communication style than intended. This becomes especially important, when taking into consideration that parents of autistic children were found to interact significantly less with their children than parents of neurotypical children in general already (Costa et al., 2019)^2^.

A review by Papoudi and colleagues (2021) including 25 studies from the US and 7 studies from the UK, mirror these findings of parents’ struggles. However, they also stress that after all, the choice of language is sometimes directed by solely pragmatic reasons, such as access to support services and professionals. Even English proficient parents reported difficulties understanding special needs terminology and medical jargon (Fox et al., 2017; You & Rosenkoetter, 2014). At the same time, it is difficult to find a set of professionals offering all the services in the respective heritage language(s). Nevertheless, all parents’ priority laid of course with the minimization of their children’s life barriers, above and beyond language choice.

## Method

### The Present Study

Previous research has analyzed the relations between bi- and multilingualism and social life in autistic people, via parent reports (see Papoudi et al., 2021, for a review) and, more recently, autistic people (Digard et al., 2020; Nolte et al., 2021). Several studies found that mono- and multilingual children on the spectrum do not show significant differences on autistic traits, a broad variety of language skills, and social, behavioral and communication outcomes (Dai et al., 2018; Gonzalez-Barrero & Nadig, 2018; Siyambalapitiya et al., 2022). Studies analyzing social skills in autistic children in multilingual environments (Papoudi et al., 2021) either report small benefits in multilingual children or cannot find any significant differences.

The studies provide data across different research methodologies (qualitative vs. quantitative, in-person vs. online designs), different sample sizes (single cases and group samples), diverse ethnic backgrounds of families (e.g., Chinese, Armenian, Canadian, Bengali, Pakistani, Armenian), and different autism diagnoses (High Functioning Autism, Asperger’s Syndrome, Pervasive Developmental Disorder – Not otherwise specified, ASD, and other unspecified diagnoses). However, the methodological soundness of the research pool has been criticized, suggesting limitations to their findings (Uljarević et al., 2016). In addition, most of the studies include either very few single cases or focus on families in predominantly English-speaking communities.

Thus, in line with the reviewed literature, the present study aims to expand the existing pool of research in the context of Europe and the many languages it has to offer. More specifically, the relationships between parents’ choice of language to communicate with their autistic child (multilingual vs. monolingual, mother tongue included vs. only foreign languages), the children’s social skills, and parents’ ability to feel comfortable, authentic, and free to express themselves when interacting with their child are analyzed. Data from 68 parents and children of 25 different nationalities, speaking 20 languages altogether, were used to investigate the following questions: (A) Are there differences between autistic children’s social skills with regard to a mono- or bi-/multilingual choice of language use?, (B) Are there differences between autistic children’s social skills with regard to language use including parents’ heritage, solely or in combination with other languages in comparison to foreign language(s) only?, and finally (C) Do parents who do not communicate with their autistic child in their own mother tongue, solely or in combination with other languages, feel less comfortable, less authentic, and cannot express themselves as freely as parents who use their mother tongue, or both their mother tongue and other foreign languages?

### Instruments and Measures

#### Questionnaire on Socio-Demographic Data

Socio-demographic data for the parents included information on their age, nationality, relation to the child, level of education, occupation, civil status, family constellation, and economic status. Socio-demographic data for the children included data on gender, age, nationality, diagnosis, intellectual development, type of schooling, academic performance, and possible comorbidities.

#### Language

Parents were asked to indicate which languages the child speaks, which languages are spoken in the home environment and in general, and which languages are used to address the child at home. Parents could either choose among suggested languages or add up to three additional languages per category. Proficiency levels were collected once for the children in languages spoken at home and once for the parents in languages used to address the child at home. Participants rated proficiencies on a 7-point Likert scale with the response possibilities of “no proficiency”, “fair proficiency”, “good proficiency”, “very good proficiency”, “fluent proficiency”, “native proficiency”, and “mother tongue”.

#### Feeling Comfortable, Authentic, and Free to Express Oneself

Regarding the language they used to interact with the child, parents were asked how comfortable, authentic, and able to express themselves they felt. Responses were assessed on a 5-point Likert scale. Response possibilities for feeling comfortable ranged from “very uncomfortable” to “very comfortable”. Response possibilities for feeling authentic ranged from “very unauthentic” to “very authentic”. Response possibilities for the question whether participants felt able to express themselves ranged from “very restrictedly” to “very freely. Each variable had a neutral central response possibility.

#### Social Skills

The social skills of the children were assessed using the Social Responsiveness Scale™, Second Edition [SRS-2] (Constantino & Gruber, 2012). This parent-report includes 65 items on a 4-point Likert scale, ranging from “not true” to “almost always true”, and aims to identify the presence and severity of social impairments in autistic people. The test provides a total score but delivers also individual scores for the five subscales “social awareness”, “social cognition”, “social communication”, “social motivation”, and “restricted interests and repetitive behavior”. Scores may range either from zero to 123 (raw scores) or 30 to 90 (t-scores), whereas higher scores indicate increased social impairment. Studies on predictive validity yielded sensitivity estimates and specificity estimates of .92 (Bruni, 2014). Internal consistency is high with Cronbach’s *α* = .95 across clinical subgroups and correlations between different age versions ranges between .94 and .96 (Bruni, 2014). Calculations for internal consistency using the present sample provided a Cronbach’s *α* = .82, indicating good internal consistency for the whole questionnaire.

#### Autistic Traits

Due to the online nature of the study and the impossibility to verify children’s ASD diagnoses, participants were asked to complete the Autism Spectrum Quotient – Children’s version questionnaire [AQ-Child] (Auyeung et al., 2008) to complement information on diagnosis. The questionnaire includes 50 items and assesses autistic traits using a 4-point Likert scale, ranging from “definitely agree” to “definitely disagree”. The test provides a total score ranging from zero to 150, with 150 representing very pronounced autistic traits. We used a cut-off score of 76, which provides a high sensitivity of .95 and specificity of .95 (Auyeung et al., 2008) to other ASD diagnostic tools. In the study by Auyeung and colleagues (2008), the questionnaire yielded a very high internal consistency, with Cronbach’s *α* = .97. In the present sample, a Cronbach’s *α* = .77 was obtained, which indicates a good internal consistency.

### Procedure

The online survey took about 25 min (*M* = 24.87, *SD* = 7.79) to be completed and was offered in German, French, and English. Parents of a 4 to 11 years old autistic child were recruited for the most part by email and posts on social media of different associations, forums, and institutions for autism (e.g. “Fondation Autisme Luxembourg”, “3AL - Autism Awareness Association Luxembourg”, and “CTSA - Centre pour enfants et jeunes présentant un trouble du spectre de l’autisme”), and professionals working with children on the spectrum.

The survey started with a brief explanation of the objective of the research project, a consent form, general instructions, and researchers’ contact information. The first part of the survey contained questions about sociodemographic data from both the parent and the autistic child. Parents were asked about their and their child’s language skills and about their feelings regarding their language use with the child (i.e. feelings of comfort, authenticity, and freedom of expression). Subsequently, the SRS-2 (Constantino & Gruber, 2012) was used to assess the social skills of the child. Participants finished by completing the AQ-Child (Auyeung et al., 2008), assessing children’s autistic traits.

Participants were offered anonymity by design. Data was subject to legal regulations, such as the German Federal Data Protection Act (Bundesdatenschutzgesetz - BCSG) and the EU General Data Protection Regulation (GDPR) and was encrypted and stored in a certified data center. The study was approved by the research ethics committees of the University of Luxembourg (ERP 20-019 SSMCA).

### Participants

From the initial 142 participants, 70 participants had to be excluded for incomplete data and four were excluded because their children did not meet the age criteria. The final sample included 68 parents (61 mothers, seven fathers) aged from 27 to 56 years (*M*_ageP_ = 39.86; *SD*_ageP_ = 6.59) reporting information on 61 male (89.7%) and seven female (10.3%) children on the spectrum, aged between 4 and 11 year old (*M*_ageC_ = 8.08; *SD*_ageC_ = 2.31). Our study population represented 25 nationalities in parents (14.71% had more than one) and 24 nationalities in children (22.06% had more than one), who spoke 20 languages altogether.

According to the parents, children were diagnosed by either a psychologist, psychiatrist, pediatrician, neurologist, or multidisciplinary team and were on average about 4 years and 8 months old (*M*_ageD_ = 4.68; *SD*_ageD_ = 2.24) when diagnosed. Frequencies of the official diagnoses and comorbidities can be retrieved from Table 1. Five children were non-verbal at the time of the survey, four used some variant of sign language, 16 were monolingual, 43 were multilingual. Regarding the AQ-Child, 60 children (88.24%) had a score above the diagnosis confidence cut-off of 76 (*M*_AQ−C_ = 94.26; *SD*_AQ−C_ = 17.99). The scores ranged from 41 to 72 for children with a score beneath the cut-off, and from 77 to 131 for children with a score above the cut-off score. Data from all children were included in the analysis to maintain the natural occurrences of autistic traits in the sample population.

**Table 1:** Frequency and within-group percentage or mean and standard-deviation values for demographic data of children in all four language groups. Pearson’s chi square (*χ*^*2*^) or One-Ways ANOVA (*F*) values for group differences, significance levels (*p*), and Cramer’s V (*V*) or eta-squared (*η*^*2*^) effect size measurements

Measure	Monolingual mother tongue *n* = 17	Monolingual foreign language *n* = 17	Multilingual mother tongue *n* = 26	Multilingual foreign languages *n* = 8	Statistics Pearson’s chi square Analysis of variance
Child Gender – Male (*n*, %)	14 (82.35)	15 (88.24)	25 (96.15)	7 (87.50)	*X*^*2*^(3, *N* = 68) = 2.25, *p* = .57, *V* = .18
Age in years (*M*, *SD*)	8.31 (1.82)	8.63 (2.08)	7.53 (2.69)	8.20 (2.35)	*F*(3, 64) = 0.88, *p* = .46, *η*^*2*^ = .04
Intelligence (*M*, *SD*)	3.08 (1.00)	3.11 (.93)	2.86 (1.03)	2.50 (1.73)	*F*(3, 35) = 0.40, *p* = .76, *η*^*2*^ = .03
Autistic traits (*M*, *SD*)	95.76 (23.22)	94.06 (11.96)	94.62 (18.21)	90.38 (18.15)	*F*(3, 64) = 0.16, *p* = .92, *η*^*2*^ = .00
Diagnosis (*n*, %)					
ASD	10 (58.82)	9 (52.94)	14 (53.85)	4 (50.00)	*χ*^*2*^(3, *N* = 68) = 0.21, *p* = .99, *V* = .06
Asperger’s syndrome	5 (29.41)	4 (23.53)	3 (11.54)	1 (12.50)	*χ*^*2*^(3, *N* = 68) = 2.57, *p* = .49, *V* = .19
Childhood autism	3 (17.65)	7 (41.18)	7 (26.92)	2 (25.00)	*χ*^*2*^(3, *N* = 68) = 2.42, *p* = .54, *V* = .19
Atypical autism	2 (11.76)	2 (11.76)	1 (3.85)	0 (0.00)	*χ*^*2*^(3, *N* = 68) = 2.08, *p* = .62, *V* = .18
High functioning autism	1 (5.88)	0 (0.00)	2 (7.69)	2 (25.00)	*χ*^*2*^(3, *N* = 68) = 5.07, *p* = .16, *V* = .27
PDD	2 (11.76)	0 (0.00)	3 (11.54)	0 (0.00)	*χ*^*2*^(3, *N* = 68) = 3.14, *p* = .44, *V* = .22
Comorbidities (*n*, %)					
Hearing disability	0 (0.00)	1 (5.88)	2 (7.69)	0 (0.00)	*χ*^*2*^(3, *N* = 68) = 1.91, *p* = .85, *V* = .17
Learning disability	5 (29.41)	10 (58.82)	7 (26.92)	2 (25.00)	*χ*^*2*^(3, *N* = 68) = 5.55, *p* = .14, *V* = .29
Language delay	10 (58.82)	12 (70.59)	19 (73.08)	6 (75.00)	*χ*^*2*^(3, *N* = 68) = 1.18, *p* = .80, *V* = .13
Other	4 (23.53)	5 (29.41)	7 (26.92)	1 (12.50)	*χ*^*2*^(3, *N* = 68) = 0.91, *p* = .87, *V* = .12

^Note: ASD = autism spectrum disorder, PDD = pervasive developmental disorder, intelligence = parent report, autistic traits = AQ−Child total raw score^

### Data Analysis

Participants were divided into four groups based on their language use. Participants who used only their mother tongue to communicate with their child were assigned to the group “monolingual mother tongue” (*n* = 17). Participants who used only a foreign language were assigned to the group “monolingual foreign language” (*n* = 17). The group “multilingual mother tongue” (*n* = 26) comprised participants who used their mother tongue and other languages. The group “multilingual foreign languages” (*n* = 8) consisted of parents who used multiple foreign languages, but not their mother tongue.

Pearson’s chi square tests and analyses of variance (One-Way ANOVAs) were calculated for group comparisons on demographic data. Bonferroni post hoc tests were applied to all tests to control for Type I error rate and Gabriel’s post-hoc tests were applied to all tests to control for sample size differences. Test results confirmed that all four groups did not differ regarding child gender, child age, child intelligence, autistic traits, and diagnoses (see Table 1). Parent gender, parent age, parent schooling level, and economic resources did not differ significantly across language groups (see Table 2). Further details on both samples can be consulted in Online Resource 1 (see Supplementary Table 1 for parents and Supplementary Table 2 for children)^3^.

**Table 2:** Frequency and within-group percentage or mean and standard-deviation values for demographic data of parents of children in all four language groups. Pearson’s chi square (*χ*^*2*^) or One-Ways ANOVA (*F*) values for group differences, significance levels (*p*), and Cramer’s V (*V*) or eta-squared (*η*^*2*^) effect size measurements

Measure	Monolingual mother tongue *n* = 17	Monolingual foreign language *n* = 17	Multilingual mother tongue *n* = 26	Multilingual foreign languages *n* = 8	Statistics Pearson’s chi square
Parent Gender – Female (*n*, %)	16 (94.12)	15 (88.24)	23 (88.46)	7 (87.50)	*X*^*2*^(3, *N* = 68) = 0.48, *p* = .95, *V* = .08
Age in years (*M*, *SD*)	39.59 (6.48)	40.21 (6.57)	40.00 (7.00)	39.38 (6.80)	*F*(3,60) = 0.04, *p* = .99, *η*^*2*^ = .00
Schooling Level (*M*, *SD*)	3.35 (0.61)	3.18 (0.73)	3.42 (0.86)	3.25 (0.71)	*F*(3,64) = 0.40, *p* = .75, *η*^*2*^ = .02
Economic resources (*M*, *SD*)	2.47 (1.18)	2.94 (1.00)	2.44 (1.04)	2.00 (1.07)	*F*(3,62) = 1.49, *p* = .23, *η*^*2*^ = .07

^Note: schooling level = parent report, economic resources = parent report^

## Results

### Are the Social Skills of Children Different Depending on the Language Used?

Analyses of variance (ANOVAs) with Bonferroni and Gabriel´s post hoc tests were used to analyze the differences in children’s social skills and the languages used to address them. No significant differences were found among groups on the SRS-2 total raw score [*F*(3,64) = 0.84, *p* = .48, *η*^*2*^ = .04], the social awareness subscale [*F*(3,64) = 0.52, *p* = .67, *η*^*2*^ = 0.02], the social cognition subscale [*F*(3,64) = 0.57, *p* = .63, *η*^*2*^ = .03], the social communication subscale [*F*(3,64) = 1.03, *p* = .39, *η*^*2*^ = .05], the social motivation subscale [*F*(3,64) = 0.98, *p* = .41, *η*^*2*^ = .04], or the restricted interests and repetitive behavior subscale [*F*(3,64) = 0.67, *p* = .57, *η*^*2*^ = .03] (see Table 3).

**Table 3:** Means (*M*), standard deviations (*SD*), *F*-values (ANOVA), significance levels (*p*), and effect sizes (*η*^*2*^*)* on the SRS-2 scores comparing the groups on children’s overall social skills (SRS-2 total raw score), social awareness (SRS-2 social awareness subscale), social cognition (SRS-2 social cognition subscale), social communication (SRS-2 social communication subscale), social motivation (SRS-2 social motivation subscale), and repetitive interests and behavior (SRS-2 repetitive interests and behavior subscale)

Measure	Monolingual mother tongue *n* = 17	Monolingual foreign language *n* = 17	Multilingual mother tongue *n* = 26	Multilingual foreign languages *n* = 8	Analysis of variance
Total score	109.00 (24.49)	105.76 (21.47)	107.69 (20.39)	94.88 (23.05)	*F*(3,64) = 0.84, *p* = .48, *η*^*2*^ = .04
Social awareness	13.76 (2.59)	13.82 (3.11)	12.77 (3.76)	13.50 (2.07)	*F*(3,64) = 0.52, *p* = .67, *η*^*2*^ = .02
Social cognition	20.35 (4.66)	20.41 (5.66)	20.69 (4.29)	18.13 (5.79)	*F*(3,64) = 0.57, *p* = .63, *η*^*2*^ = .03
Social communication	34.88 (9.32)	34.24 (8.34)	35.69 (7.78)	29.88 (7.38)	*F*(3,64) = 1.03, *p* = .39, *η*^*2*^ = .05
Social motivation	18.18 (6.42)	17.82 (3.83)	18.27 (3.66)	15.13 (5.25)	*F*(3,64) = 0.98, *p* = .41, *η*^*2*^ = .04
Restricted interests and repetitive behavior	21.82 (7.32)	19.47 (6.53)	20.27 (5.55)	18.25 (7.32)	*F*(3,64) = 0.67, *p* = .57, *η*^*2*^ = .03

Additional independent samples *t*-tests did not show significant differences for comparison between mono- vs. multilingual language use, between mother tongue vs. only foreign language(s), between “monolingual mother tongue” vs. all other groups and “monolingual foreign language” vs all other groups. Exact values can be consulted in Online Resource 2 (see Supplementary Table 3). Analyses of covariance (ANCOVAs) controlling for demographic variables such as gender, age, nationality, economic resources, intelligence, autistic traits, diagnoses, and comorbidities of children were conducted. Differences between children groups remained insignificant. Calculations can be consulted in Online Resource 3 (see Supplementary Table 4).

### Do Parents Feel Differently When Communicating with Their Children Depending on the Language Used?

**Fig. 1:**
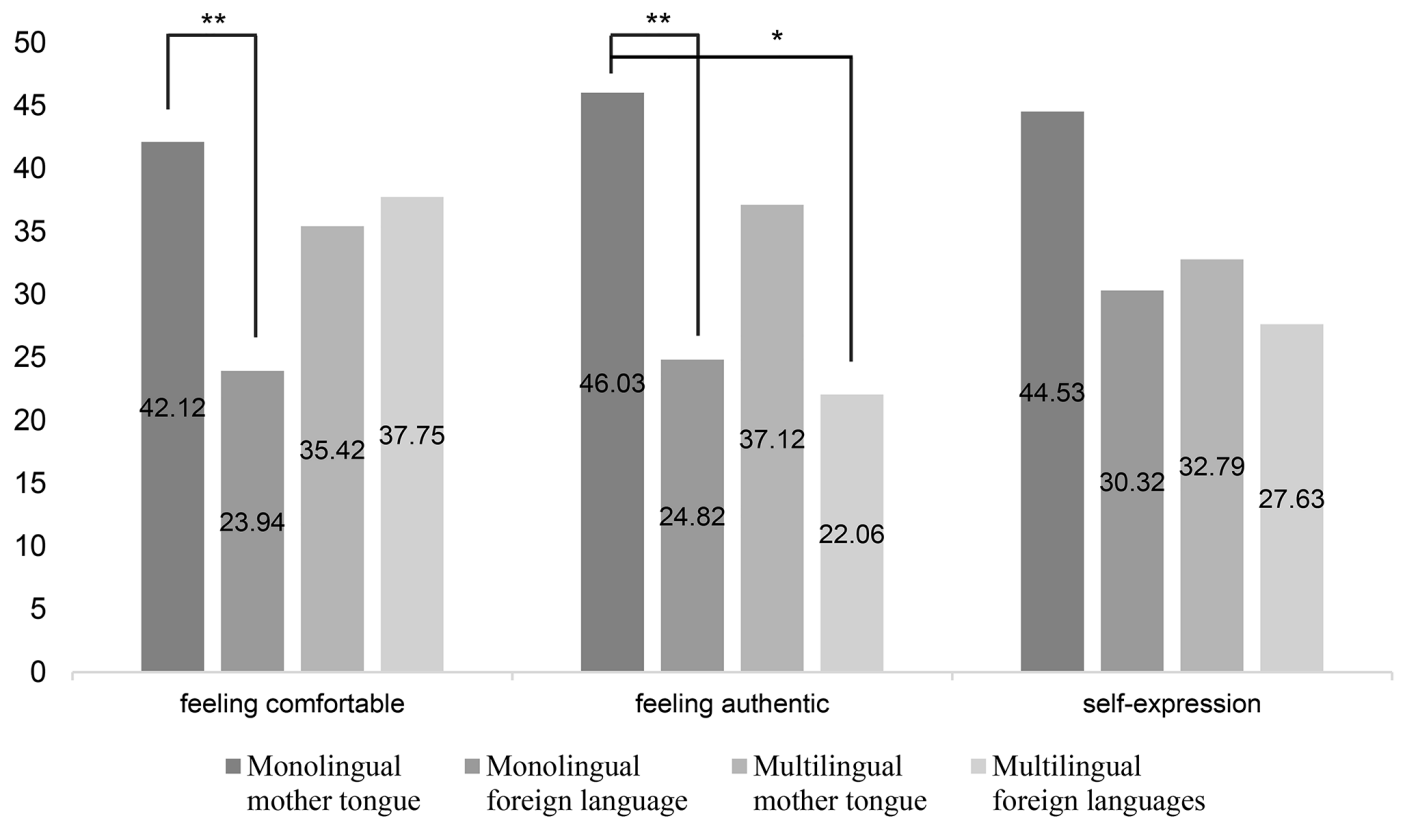
Mean ranks, obtained by pairwise comparisons of feeling comfortable, feeling authentic, and self-expression in parents regarding their use of language with their child. Note: * *p* < .05, ** *p* < .01

Because the variables feeling comfortable, authentic, and able to express oneself freely did not meet the assumption of normality, the non-parametric Kruskal-Wallis test was used to assess differences depending on the language use with their children. Test results showed significant group differences for feeling comfortable [*H*(3) = 11.23, *p* = .01] and authentic [*H*(3) = 16.73, *p* = .001]. Group differences for self-expression were marginally significant [*H*(3) = 7.60, *p* = .055]. Pairwise comparisons with Bonferroni corrected *p*-values (see Fig. 1) showed that “monolingual mother tongue” parents felt significantly more comfortable [*H*(3) = 18.18, *p* < .01, *r* = .39] than “monolingual foreign language” parents when addressing their child. In addition, “monolingual mother tongue” parents felt also significantly more authentic than “monolingual foreign language” parents [*H*(3) = 21.21, *p* < .01, *r* = .42] and “multilingual foreign languages” parents [*H*(3) = 23.97, *p* < .05, *r* = .38]. No significant group differences were found for self-expression. Additional Mann-Whitney tests for detailed group comparisons can be retrieved from Online Resource 4 and Supplementary Figs. 1–4.

Subsequently, Spearman correlations have been calculated in addition to analyze the relationship between parents’ language proficiency in the language used to address their children and the variables feeling comfortable, authentic, and able to express oneself freely. Parents’ language proficiency correlated significantly with feeling authentic (*r*_*s*_ = .374, 95% BCa CI [.142, .567], *p* < .01) and being able to express oneself freely (*r*_*s*_ = .377, 95% BCa CI [.145, .569], *p* < .01). The relation to feeling comfortable was marginally non-significant (*r*_*s*_ = .225, 95% BCa CI [-.021, .446], *p* = .065).

## Discussion

### Language Use and Social Skills

Children’s social skills were not found to be related to the language(s) used by parents when interacting with their children. Group differences remained insignificant even when controlling for children’s demographic aspects such as gender, age, nationality, economic resources, intelligence, autistic traits, diagnoses, and comorbidities. Thus, similar to previous studies, our findings do not identify any indication that multilingualism harms autistic children’s social skills (Hambly & Fombonne, 2012; Li et al., 2017; Ohashi et al., 2012; Reetzke et al., 2015; Siyambalapitiya et al., 2022). Our findings are for the most part concordant with previous research (Papoudi et al., 2021). However, we also do not find any significant advantages of multilingualism, as some other studies report (Beauchamp et al., 2023; Valicenti-McDermott et al., 2012; Wharton et al., 2000; Yu, 2016).

Since we did not run comparisons in relation to the languages children were exposed to outside their home environment, it could have been the case that parents would only use one language (e.g. their mother tongue) at home, but that the child would be exposed to other languages at school or leisure activities. Among our 68 participants, 34 reported using only one language with their child. Meanwhile, 20 children were reported to be monolingual themselves. However, out of those 20, only 12 were addressed in one language at home. Thus, 58 participants were either exposed to several languages at home or were proficient in more than one language despite a monolingual upbringing at home. Previous research found that any regular exposure to a second language, beyond whether parents use one or several languages at home, may suffice to increase theory of mind in communication (Fan et al., 2015; Liberman et al., 2017). Thus, the children that were assigned to the monolingual groups due to their parents’ language use at home might have be growing up multilingual nevertheless and have already benefited from increased social communication skills.

### Language Use and Parents’ Ability to Feel Free, Comfortable, and Express Themselves Freely

When comparing all four language groups against each other, parents who used only one foreign language felt significantly less comfortable and less authentic than parents who used only their mother tongue. Parents who used multiple foreign languages felt significantly less authentic than parents who used only their mother tongue. In addition, parents’ proficiency in the language used to address their child at home correlated significantly with feeling authentic and able to express oneself freely, and marginally with feeling comfortable.

Our results suggest that parents feel the most comfortable, authentic, and able to express themselves freely when using their mother tongue and/or being proficient in the language that they use with their child. Consequently, they experience the most difficulties when omitting their mother tongue. These findings are comparable to the findings by previous studies (see Papoudi et al., 2021, for a review). Parents who do not use their mother tongue might experience miscommunication, mistakes with translation, and resulting frustration (Jegatheesan, 2011; Kremer-Sadlik, 2005; Yu, 2016). It is therefore no surprise that parents were observed to be less affectionate with their children in foreign languages than in their mother tongue (Wharton et al., 2000). Parents might simply not have enough proficiency in the recommended language to express their affections, humor, and personality and to engage with their children as freely as in their mother tongue (Yu, 2016). In addition, parents reported as well that it would be very difficult to share their heritage culture with their children, if they were to neglect their mother tongue (Jegatheesan, 2011). Parents’ cultural identity, songs, or stories that cannot be translated, might not be passed on to their children.

If parents would engage less with their children due to communication issues or feeling unwell when doing so, it could have an indirect effect on children’s social skills. Since children learn social skills, among others, through their parents (Ladd, 2005), restricted parental engagement could provide less opportunities for children to learn appropriate social skills, and to other aspects of child development of course. We suggest that future research investigates the possibility of an interplay between parental language use, parental language proficiency, and children’s social skills via covariation, moderation, or mediation analyses.

### Limitations

Due to the online study design, children’s diagnoses, as well as intellectual, language and social abilities could not be assessed by researchers directly. Since we included children of all ages and range of autistic traits, some children would not have been able to report for themselves. Parent reports allowed us to assess a more diverse pool of autistic people. However, the subjectiveness of the parents’ responses as well as the lack of self-advocate data creates an overall limitation to our study. Data quality relies on the accuracy of parent estimations regarding their children’s abilities and would be substantially increased by in person data collection and additional self-advocate data. We recommend that future research consider this in their study designs and that they might use more elaborate measures such as the second edition of the Autism Diagnostic Observation Schedule™ [ADOS®-2] (Lord et al., 2012) and the Social Skills Improvement System SSIS Rating Scales (Gresham & Elliot, 2008) and non-verbal language tests, such as the EVT-3 (Williams, 2018) and PPVT-5 (Dunn, 2019) for example.

It has been shown that parents are reliable informants on their children’s language proficiencies (Bedore et al., 2011). Rating language proficiencies for bilingual children (English and Spanish), parents’ ratings correlated significantly with their child’s performance in standardized language tests. Nevertheless, parent reports on language proficiency should be treated with caution, as we questioned always only one parent. However, 66 participants (97.06%) reported at least one additional family member to currently live with the child, who may have different proficiencies and hence could have provided different results to our analyses.

Furthermore, it is important to note that despite the many nationalities and ethnicities within our participants, most of them are of European origin and would thus be considered White or Caucasian. In addition, 86.8% of the participants had secondary school diploma or higher. These overrepresentations may impact the interpretation of results. Similarly, we have not assessed whether parents were autistic themselves, which could have influences their responses importantly.

Lastly, future research should use a longitudinal design, allowing to track the development of children’s social and language abilities, parent’s language use, and parent’s feelings over time. It would allow for more in depth analysis of the relations between the different constructs and provide a more comprehensive assessment of the impact of language use on both outcomes.

### Implications

Although the results of this study should be interpreted with caution and he connections between language use and other aspects of child development have not been explored in this study, the present findings offer some implications for the support of families with autistic children regarding language use. As social skills of autistic children did not differ significantly among one or several languages, or any combination of mother tongue and foreign languages, we advise families to opt for a multilingual language use if relevant for them. Considering previous research indicating no negative outcomes on autistic traits, language skills, and cognitive and executive functions regarding multilingualism in autistic children either (see Uljarević et al., 2016, for a review), we do not find any reason as to why parents should raise their children monolingually if they wish to do differently. On the contrary, parents as well as autistic self-advocates have identified several positive outcomes such as increased social interactions, increased educational and employment opportunities, as well as positive psychological outcomes (Howard et al., 2021; Kay-Raining Bird et al., 2012; Nolte et al., 2021).

In addition, our results and previous findings (e.g. Howard et al., 2021; Ijalba, 2016) indicate negative outcomes on parents’ interaction with their child if they are to neglect their mother tongue and use foreign language(s). These findings suggest that advising parents to discard their mother tongue and use culturally predominant language, for example, cannot be supported scientifically and may even cause harm for parents and the overall family dynamic. This gains even more importance, when considering that parents of autistic children were found to interact significantly less with their children than parents of neurotypical children in general already (Costa et al., 2019).

Our findings may encourage specialists to acknowledge the importance of parents’ familiarity with the language they use with their child. Parents should be able to interact with their children at ease. In addition, potential benefits and absence of harms of multilingualism for both parents and children should not be ignored. This is especially important for environments where multilingualism is almost inevitable, such as countries with multiple official languages. Advising parents to raise their child multilingually may facilitate access to therapeutic treatment, childcare, and social interaction in the multicultural society and family, and thus improve support and orientation for families with a child on the spectrum.

## Conclusions

Despite the limitations, the present study provides important insight on the relationship between language use and social skills in autistic children. Our findings suggest that children’s social skills are not related to parents’ language use with them. However, if parents do not use their mother tongue and/or if they are not proficient in the used language(s), they may be at increased risk of feeling less comfortable, authentic, and able to express themselves freely when interacting with their child. This might lead to subsequent difficulties in family interaction. Thus, professionals advising parents on their language use with their autistic children should take into account all advantages and disadvantages to different language use combinations and mind the possible necessity and individual relevance of multilingualism.

## Electronic Supplementary Material

Below is the link to the electronic supplementary material.

Supplementary Material 1

Supplementary Material 2

Supplementary Material 3

Supplementary Material 4

## Footnotes

1 This article uses the terminologies *autism*, *autistic*, and *on the spectrum* in acknowledgement to the findings of empirical studies on stakeholders’ perspectives and preferences regarding the terminology, used to communicate about autism (Buijsman et al., 2023; Bury et al., 2023; Kenny et al., 2016; Lei at al., 2021).

2 It is of course important to note, that there is a vast interplay of factors to be considered in the parents’ interaction with their autistic children (e.g., reduced joined attention [Adamson et al., 2012], autistic traits and difficulties in communication [Beurkens et al., 2013], parenting style [Riany et al., 2017], …) and that, in general, emotion-related socialization is influenced not only by parents’ personality and cultural influences, but also by their children’s characteristics (Eisenberg et al., 1998).

3 In the additional analyses, the four groups have been combined in pairs and or different group combinations. Group comparison analyses remained insignificant in all combinations for all analyzed demographic and descriptive variables (child gender, age, presence of intellectual developmental delay, autistic traits, and diagnoses, as well as parent gender, age, schooling level, economic resources).
