# Correction: Should Parents Only Use One Language with Their Autistic Children? The Relations between Multilingualism, Children‘s Social Skills, and Parent-Child Communication

**DOI:** 10.1007/s10803-024-06465-5

**Published:** 2024-07-31

**Authors:** Maïte Franco, Andreia P. Costa

**Affiliations:** Institute for Health and Behaviour, Department of Behavioural and Cognitive Sciences, Maison des Sciences Humaines, University of Luxembourg, 11 Porte des Sciences, Esch-sur-Alzette, L-4366 Luxembourg

**Correction: Journal of Autism and Developmental Disorders**

10.1007/s10803-024-06347-w

The article “Should Parents Only Use One Language with Their Autistic Children? The Relations Between Multilingualism, Children’s Social Skills, and Parent-Child Communication”, written by Maïte Franco and Andreia P. Costa was originally published electronically on the publisher’s internet portal on 29th May 2024 without open access. With the author(s)’ decision to opt for Open Choice the copyright of the article changed on 27th June 2024 to © The Author(s) 2024 and the article is forthwith distributed under a Creative Commons Attribution 4.0 International License, which permits use, sharing, adaptation, distribution and reproduction in any medium or format, as long as you give appropriate credit to the original author(s) and the source, provide a link to the Creative Commons licence, and indicate if changes were made. The images or other third party material in this article are included in the article’s Creative Commons licence, unless indicated otherwise in a credit line to the material. If material is not included in the article’s Creative Commons licence and your intended use is not permitted by statutory regulation or exceeds the permitted use, you will need to obtain permission directly from the copyright holder. To view a copy of this licence, visit http://creativecommons.org/licenses/by/4.0.

