# Supplementary material for: Should Parents Only Use One Language with Their Autistic Children? The Relations Between Multilingualism, Children‘s Social Skills, and Parent-Child Communication
Source: J Autism Dev Disord. 2024 May 29;55(8):2761–73. doi: 10.1007/s10803-024-06347-w (PMC12296994; doi:10.1007/s10803-024-06347-w)
Supplement: Supplementary file 1 — Supplementary Material 1 [file 10803_2024_6347_MOESM1_ESM.docx]

**Supplementary Table 1**. Frequency and within-group percentages or mean and standard-deviation values for supplemental demographic data of parents of children in all four language groups. Pearson’s chi square (*χ*^2^) values for group differences, significance levels (*p*), and Cramer’s V (*V*) effect size measurements.

| Measure | monolingual mother tongue  *n* = 17 | monolingual foreign language  *n* = 17 | multilingual with mother tongue  *n* = 26 | multilingual without mother tongue  *n* = 8 | Statistics  Pearson’s chi square |
| --- | --- | --- | --- | --- | --- |
| Parent Nationality (*n*, %)  **Luxembourgish**  German  French  Belgian  Portuguese  Italian  **Other** | **0 (0%)**  6 (35.3%)  4 (23.5%)  2 (11.8%)  2 (11.8%)  1 (5.9%)  **3 (17.6%)** | **4 (23.5%)**  2 (11.8%)  1 (5.9%)  0 (0%)  2 (11.8%)  3 (17.6%)  **10 (58.8%)** | **1 (3.8%)**  4 (15.4%)  5 (19.2%)  1 (3.8%)  1 (3.8%)  1 (3.8%)  **17 (65.4%)** | **0 (0%)**  0 (0%)  1 (12.5%)  2 (25%)  1 (12.5%)  2 (25%)  **2 (25%)** | ***χ*^2^(3, *N* = 68) = 8.98, *p* < .05, *V* = .36**  *χ*^2^(3, *N* = 68) = 5.85, *p* = .13, *V* = .29  *χ*^2^(3, *N* = 68) = 2.27, *p* = .58, *V* = .18  *χ*^2^(3, *N* = 68) = 5.96, *p* = .08, *V* = .30  *χ*^2^(3, *N* = 68) = 1.30, *p* = .78, *V* = .14  *χ*^2^(3, *N* = 68) = 4.40, *p* = .28, *V* = .25  ***χ*^2^(3, *N* = 68) = 11.92, *p* < .01, *V* = .42** |
| Occupation (*n*, %)  Unemployed  In training / schooling  Maternity / parental leave  Part-time employed  Full-time employed  Other | 4 (23.5%)  0 (0%)  1 (5.9%)  7 (41.2%)  3 (17.6%)  2 (11.8%) | 6 (35.3%)  0 (0%)  1 (5.9%)  5 (29.4%)  4 (23.5%)  1 (5.9%) | 7 (26.9%)  2 (7.7%)  2 (7.7%)  7 (26.9%)  5 (19.2%)  3 (11.5%) | 2 (25%)  0 (0%)  1 (12.5%)  1 (12.5%)  3 (37.5%)  1 (12.5%) | *χ*^2^(3, *N* = 68) = .67, *p* = .91, *V* = .10  *χ*^2^(3, *N* = 68) = 3.33, *p* = .49, *V* = .22  *χ*^2^(3, *N* = 68) = .42, *p* = 1.00, *V* = .08  *χ*^2^(3, *N* = 68) = 2.31, *p* = .53, *V* = .18  *χ*^2^(3, *N* = 68) = 1.44, *p* = .72, *V* = .15  *χ*^2^(3, *N* = 68) = .48, *p* = .95, *V* = .08 |
| Marital status (*n*, %)  Single  Partnership  Civil union / Married | 3 (17.6%)  1 (5.9%)  13 (76.5%) | 2 (11.8%)  3 (17.6%)  12 (70.6%) | 3 (11.5%)  4 (15.4%)  19 (73.1%) | 0 (0%)  2 (25%)  6 (75%) | *χ*^2^(3, *N* = 68) = 1.64, *p* = .71, *V* = .16  *χ*^2^(3, *N* = 68) = 1.86, *p* = .67, *V* = .17  *χ*^2^(3, *N* = 68) = .16, *p* = 1.00, *V* = .05 |

**Supplementary Table 2**. Frequency and within-group percentages or mean and standard-deviation values for supplemental demographic data of children in all four language groups. Pearson’s chi square (*χ*^2^) or One-Ways ANOVA (*F*) values for group differences, significance levels (*p*), and Cramer’s V (*V*) or eta-squared (*η^2^*) effect size measurements.

| Measure | monolingual mother tongue  *n* = 17 | monolingual foreign language  *n* = 17 | multilingual with mother tongue  *n* = 26 | multilingual without mother tongue  *n* = 8 | Statistics  Pearson’s chi square  Analysis of variance |
| --- | --- | --- | --- | --- | --- |
| Age when diagnosed in years (*M*, *SD*) | 5.40 (2.17) | 4.71 (2.22) | 4.29 (2.11) | 4.35 (2.80) | *F*(3,64) = .92, *p* = .44, *η^2^* = .04 |
| Child Nationality (*n*, %)  Luxembourgish  German  French  Belgian  Portuguese  Italian  **Other** | 1 (5.9%)  6 (35.3%)  5 (29.4%)  2 (11.8%)  2 (11.8%)  0 (0%)  **4 (23.5%)** | 5 (29.4%)  4 (23.5%)  1 (5.9%)  0 (0%)  0 (0%)  1 (5.9%)  **7 (41.2%)** | 2 (7.7%)  5 (19.2%)  4 (15.4%)  1 (3.8%)  1 (3.8%)  1 (3.8%)  **20 (76.9%)** | 0 (0%)  0 (0%)  2 (25%)  2 (25%)  1 (12.5%)  2 (25%)  **3 (37.5%)** | *χ*^2^(3, *N* = 68) = 7.15, *p* = .07, *V* = .32  *χ*^2^(3, *N* = 68) = 4.14, *p* = .28, *V* = .25  *χ*^2^(3, *N* = 68) = 3.63, *p* = .33, *V* = .23  *χ*^2^(3, *N* = 68) = 5.96, *p* = .08, *V* = .30  *χ*^2^(3, *N* = 68) = 2.95, *p* = .45, *V* = .21  *χ*^2^(3, *N* = 68) = 6.54, *p* = .10, *V* = .31  ***χ*^2^(3, *N* = 68) = 13.33, *p* < .01 *V* = .44** |
| Schooling (*n*, %)  Public Schooling  Private Schooling  Special Education  Private Lessons  Other  Speech Therapist | 10 (58.8%)  0 (0%)  4 (23.5%)  1 (5.9%)  3 (17.6%)  8 (47.1%) | 6 (35.3%)  0 (0%)  8 (47.1%)  1 (5.9%)  1 (5.9%)  10 (58.8%) | 18 (69.2%)  0 (0%)  7 (26.9%)  2 (7.7%)  0 (0%)  12 (46.2%) | 3 (37.5%)  1 (12.5%)  5 (62.5%)  0 (0%)  0 (0%)  4 (50%) | *χ*^2^(3, *N* = 68) = 5.86, *p* = .13, *V* = .29  *χ*^2^(3, *N* = 68) = 7.61, *p* = .12, *V* = .34  *χ*^2^(3, *N* = 68) = 5.45, *p* = .15, *V* = .28  *χ*^2^(3, *N* = 68) = .65, *p* = 1.00, *V* = .10  *χ*^2^(3, *N* = 68) = 6.38, *p* = .13, *V* = .31  *χ*^2^(3, *N* = 68) = .74, *p* = .92, *V* = .10 |
| Academic success (*M*, *SD*) | 3.53 (1.23) | 3.00 (1.32) | 3.08 (1.32) | 4.13 (1.13) | *F*(3,64) = 1.86, *p* = .15, *η^2^* = .08 |
| Languages (*n*, %)  Non-verbal  Sign language  Luxembourgish  German  French  English  Portuguese  Italian  Other | 1 (5.9%)  0 (0%)  3 (17.6%)  6 (35.3%)  9 (52.9%)  5 (29.4%)  1 (5.9%)  0 (0%)  6 (35.3%) | 1 (5.9%)  1 (5.9%)  5 (29.4%)  9 (52.9%)  6 (35.3%)  8 (47.1%)  3 (17.6%)  1 (5.9%)  7 (41.2%) | 7 (26.9%)  3 (11.5%)  3 (11.5%)  7 (26.9%)  8 (30.8%)  12 (46.2%)  2 (7.7%)  1 (3.8%)  8 (30.8%) | 0 (0%)  0 (0%)  0 (0%)  4 (50%)  6 (75%)  5 (62.5%)  1 (12.5%)  2 (25%)  4 (50%) | *χ*^2^(3, *N* = 68) = 7.06, *p* = .06, *V* = .32  *χ*^2^(3, *N* = 68) = 3.07, *p* = .38, *V* = .21  *χ*^2^(3, *N* = 68) = 4.18, *p* = .26, *V* = .25  *χ*^2^(3, *N* = 68) = 3.50, *p* = .34, *V* = .23  *χ*^2^(3, *N* = 68) = 6.04, *p* = .12, *V* = .30  *χ*^2^(3, *N* = 68) = 2.69, *p* = .44, *V* = .20  *χ*^2^(3, *N* = 68) = 1.59, *p* = .77, *V* = .15  *χ*^2^(3, *N* = 68) = 6.54, *p* = .10, *V* = .31  *χ*^2^(3, *N* = 68) = 2.26, *p* = .52, *V* = .18 |

*^Note^*^: academic success = parent report^
