# Supplementary material for: Should Parents Only Use One Language with Their Autistic Children? The Relations Between Multilingualism, Children‘s Social Skills, and Parent-Child Communication
Source: J Autism Dev Disord. 2024 May 29;55(8):2761–73. doi: 10.1007/s10803-024-06347-w (PMC12296994; doi:10.1007/s10803-024-06347-w)
Supplement: Supplementary file 2 — Supplementary Material 2 [file 10803_2024_6347_MOESM2_ESM.docx]

**Supplementary table 3**. Independent samples t-tests for differences between language groups regarding the social skills of the children. T-Test (*t*) values, significance levels (*p*), and effect sizes (*r*) for group comparisions on children’s overall social skills (SRS-2 total raw score), social awareness (SRS-2 social awareness subscale), social cognition (SRS-2 social cognition subscale), social communication (SRS-2 social communication subscale), social motivation (SRS-2 social motivation subscale), and repetitive interests and behavior (SRS-2 repetitive interests and behavior subscale).

| Measure | monolingual mother  tongue vs. all other language groups | monolingual foreign  language vs. all other language groups | monolingual vs. multilingual language groups | mother tongue vs. foreign language groups |
| --- | --- | --- | --- | --- |
| Total score | *t*(66) = .64, *p* = .52, *r* = .01 | *t*(66) = -.06, *p* = .96, *r* = .01 | *t*(66) = .51, *p* = .62, *r* = .06 | *t*(66) = 1.08, *p* = .27, *r* = .13 |
| Social awareness | *t*(66) = .60, *p* = .55, *r* = .07 | *t*(66) = .69, *p* = .49, *r* = .08 | *t*(66) = 1.12, *p* = .27, *r* = .14 | *t*(66) = -.70, *p* = .48, *r* = .09 |
| Social cognition | *t*(66) = .11, *p* = .91, *r* = .01 | *t*(66) = .17, *p* = .87, *r* = .02 | *t*(66) = .25, *p* = .81, *r* = .03 | *t*(66) = .71, *p* = .48, *r* = .09 |
| Social communication | *t*(66) = .25, *p* = .80, *r* = .03 | *t*(66) = -.12, *p* = .91, *r* = .01 | *t*(66) = .12, *p* = .91, *r* = .01 | *t*(66) = 1.22, *p* = .23, *r* = .15 |
| Social motivation | *t*(20.44) = .33, *p* = .74, *r* = .07 | *t*(66) = .06, *p* = .95, *r* = .01 | *t*(66) = .41, *p* = .68, *r* = .05 | *t*(66) = 1.08, *p* = .29, *r* = .13 |
| Restricted interests  and repetitive behavior | *t*(66) = 1.19, *p* = .24, *r* = .15 | *t*(66) = -.55, *p* = .58, *r* = .07 | *t*(66) = .54, *p* = .59, *r* = .07 | *t*(66) = 1.12, *p* = .27, *r* = .14 |
