# Supplementary material for: Should Parents Only Use One Language with Their Autistic Children? The Relations Between Multilingualism, Children‘s Social Skills, and Parent-Child Communication
Source: J Autism Dev Disord. 2024 May 29;55(8):2761–73. doi: 10.1007/s10803-024-06347-w (PMC12296994; doi:10.1007/s10803-024-06347-w)
Supplement: Supplementary file 3 — Supplementary Material 3 [file 10803_2024_6347_MOESM3_ESM.docx]

**Supplementary table 4**. Analyses of covariance (ANCOVAs) between social skills and languages use, controlling for demographic data such as gender, age, nationality, economic resources, intelligence, autistic traits, diagnoses, and comorbidities of children. ANCOVA (*F*) values for group differences, significance levels (*p*), and adjusted R squared (*r^2^*) effect size measurements.

| Measure | SRS-2 total raw score  ANCOVA (*F*) | Social awareness  ANCOVA (*F*) | Social cognition  ANCOVA (*F*) | Social communication  ANCOVA (*F*) | Social motivation  ANCOVA (*F*) | restricted interests and repetitive behavior  ANCOVA (*F*) |
| --- | --- | --- | --- | --- | --- | --- |
| Gender | *F*(3,63) = 0.83, *p* = .48, *r^2^* = -.02 | *F*(3,63) = 0.59, *p* = .62, *r^2^* = -.03 | *F*(3,63) = 0.59, *p* = .62, *r^2^* = -.03 | *F*(3,63) = 1.00, *p* = .40, *r^2^* = -.01 | *F*(3,63) = 1.20, *p* = .32, *r^2^* = .06 | *F*(3,63) = 0.67, *p* = .58, *r^2^* = -.03 |
| Age | *F*(3,63) = 0.87, *p* = .46, *r^2^* = -.01 | *F*(3,63) = 0.57, *p* = .64, *r^2^* = -.03 | *F*(3,63) = 0.67, *p* = .58, *r^2^* = -.01 | *F*(3,63) = 1.07, *p* = .37, *r^2^* = -.01 | *F*(3,63) = 1.05, *p* = .38, *r^2^* = .00 | *F*(3,63) = 0.67, *p* = .57, *r^2^* = -.03 |
| Nationality | *F*(3,63) = 0.89, *p* = .45, *r^2^* = -.00 | *F*(3,63) = 1.06, *p* = .37, *r^2^* = .02 | *F*(3,63) = 0.61, *p* = .61, *r^2^* = -.01 | *F*(3,63) = 1.03, *p* = .39, *r^2^* = .02 | *F*(3,63) = 1.06, *p* = .37, *r^2^* = .02 | *F*(3,63) = 0.67, *p* = .58, *r^2^* = -.03 |
| Economic resources | *F*(3,61) = 0.90, *p* = .45, *r^2^* = -.02 | *F*(3,61) = 0.54, *p* = .66, *r^2^* = -.04 | *F*(3,61) = 0.63, *p* = .60, *r^2^* = -.03 | *F*(3,61) = 0.94, *p* = .43, *r^2^* = -.02 | *F*(3,61) = 1.28, *p* = .29, *r^2^* = .02 | *F*(3,61) = 0.79, *p* = .51, *r^2^* = -.03 |
| Intelligence | *F*(3,34) = 0.23, *p* = .88, *r^2^* = -.07 | *F*(3,34) = 0.78, *p* = .51, *r^2^* = -.04 | *F*(3,34) = 0.20, *p* = .89, *r^2^* = -.08 | *F*(3,34) = 0.45, *p* = .72, *r^2^* = -.06 | *F*(3,34) = 0.39, *p* = .76, *r^2^* = .02 | *F*(3,34) = 0.05, *p* = .98, *r^2^* = -.11 |
| Autistic traits | *F*(3,63) = 0.73, *p* = .54, *r^2^* = .40 | *F*(3,63) = 0.61, *p* = .61, *r^2^* = .09 | *F*(3,63) = 0.48, *p* = .70, *r^2^* = .33 | *F*(3,63) = 0.93, *p* = .43, *r^2^* = .27 | *F*(3,63) = 0.88, *p* = .46, *r^2^* = .31 | *F*(3,63) = 0.54, *p* = .66, *r^2^* = .19 |
| Diagnosis | *F*(3,63) = 0.80, *p* = .50, *r^2^* = .00 | *F*(3,63) = 0.47, *p* = .71, *r^2^* = -.03 | *F*(3,63) = 0.57, *p* = .64, *r^2^* = -.03 | *F*(3,63) = 1.02, *p* = .39, *r^2^* = -.00 | *F*(3,63) = 0.97, *p* = .42, *r^2^* = .01 | *F*(3,63) = 0.60, *p* = .62, *r^2^* = .01 |
| Comorbidities |  |  |  |  |  |  |
| Hearing difficulties | *F*(3,63) = 0.79, *p* = .50, *r^2^* = -.01 | *F*(3,63) = 0.65, *p* = .59, *r^2^* = -.01 | *F*(3,63) = 0.48, *p* = .70, *r^2^* = -.01 | *F*(3,63) = 0.90, *p* = .45, *r^2^* = .01 | *F*(3,63) = 0.89, *p* = .45, *r^2^* = .01 | *F*(3,63) = 0.64, *p* = .59, *r^2^* = -.02 |
| Learning difficulties | *F*(3,63) = 0.87, *p* = .46, *r^2^* = -.00 | *F*(3,63) = 0.43, *p* = .73, *r^2^* = -.03 | *F*(3,63) = 0.58, *p* = .63, *r^2^* = .00 | *F*(3,63) = 1.06, *p* = .37, *r^2^* = .00 | *F*(3,63) = 0.98, *p* = .41, *r^2^* = -.00 | *F*(3,63) = 0.71, *p* = .55, *r^2^* = -.03 |
| Language  delay | *F*(3,63) = 0.81, *p* = .49, *r^2^* = -.02 | *F*(3,63) = 0.48, *p* = .70, *r^2^* = -.03 | *F*(3,63) = 0.58, *p* = .63, *r^2^* = -.03 | *F*(3,63) = 1.00, *p* = .40, *r^2^* = -.01 | *F*(3,63) = 0.99, *p* = .40, *r^2^* = -.01 | *F*(3,63) = 0.63, *p* = .60, *r^2^* = -.03 |
| Other comorbidities | *F*(3,63) = 0.72, *p* = .55, *r^2^* = .01 | *F*(3,63) = 0.52, *p* = .67, *r^2^* = -.03 | *F*(3,63) = 0.47, *p* = .71, *r^2^* = -.01 | *F*(3,63) = 0.91, *p* = .44, *r^2^* = .00 | *F*(3,63) = 0.72, *p* = .55, *r^2^* = .01 | *F*(3,63) = 0.65, *p* = .59, *r^2^* = -.00 |

*^Note^*^: economic resources = parent report, intelligence = parent report, autistic traits = AQ-Child total raw score^
