# Supplementary material for: Should Parents Only Use One Language with Their Autistic Children? The Relations Between Multilingualism, Children‘s Social Skills, and Parent-Child Communication
Source: J Autism Dev Disord. 2024 May 29;55(8):2761–73. doi: 10.1007/s10803-024-06347-w (PMC12296994; doi:10.1007/s10803-024-06347-w)

**Supplementary Fig. 1** Mann-Whitney tests (mean ranks) comparing parents who used only their mother tongue to all other parents (only a foreign language, their mother tongue and foreign languages, multiple foreign languages) regarding feeling comfortable, authentic, and free to express themselves when interacting with their child. *Note*: **p* < .05, ***p* < .01


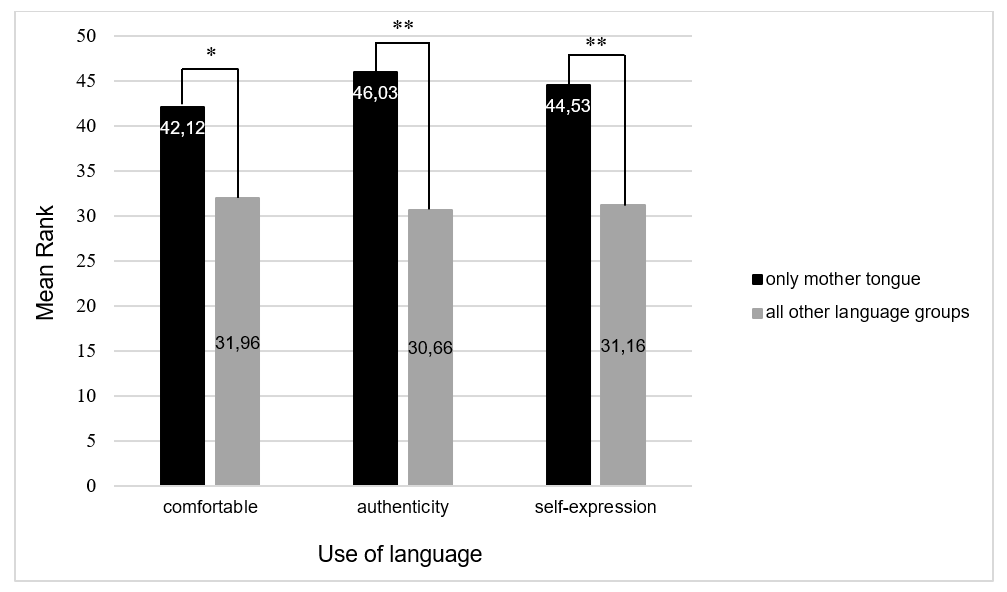


**Supplementary Fig. 2** Mann-Whitney tests (mean ranks) comparing parents who used only one foreign language to all other parents (only their mother tongue, their mother tongue and foreign languages, multiple foreign languages) regarding feeling comfortable, authentic, and free to express themselves when interacting with their child. *Note*: * *p* < .05, ** *p* < .01


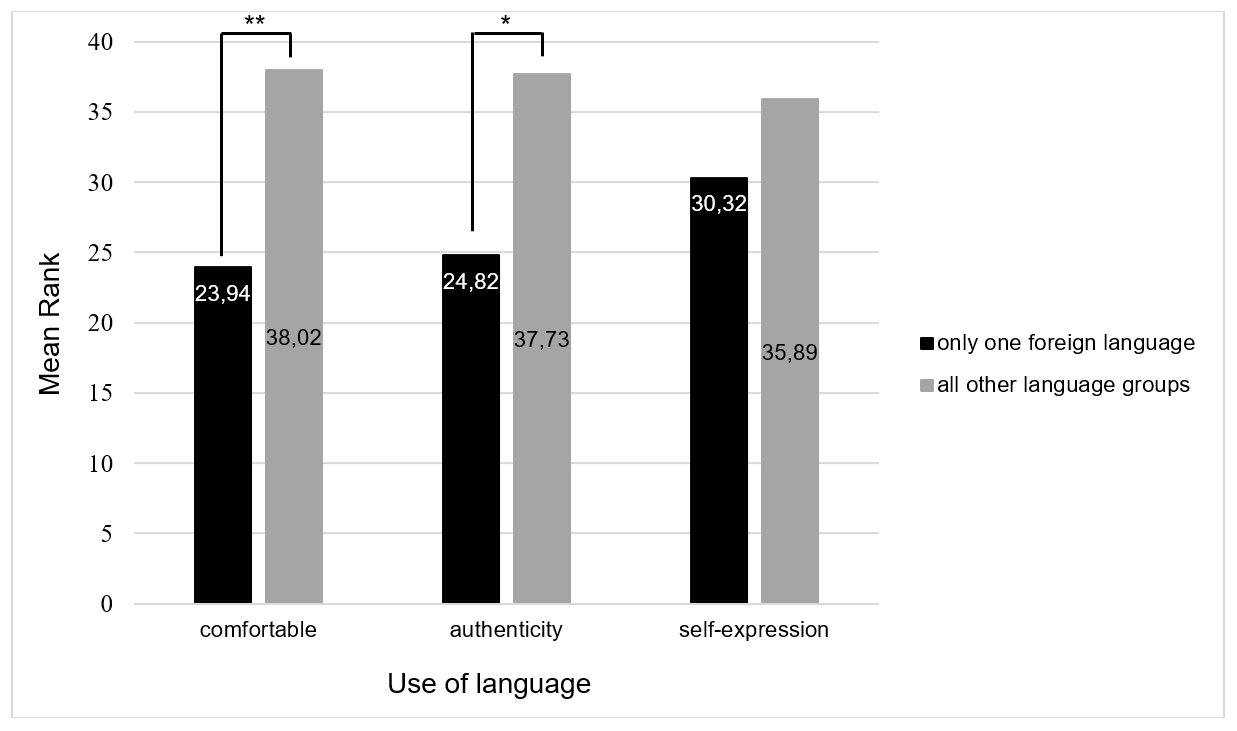


**Supplementary Figure 3***.* Mann-Whitney tests (mean ranks) comparing parents who used only one language with their child to parents who used more than one language, regardless of whether it was their mother tongue or a foreign language, regarding feeling comfortable, authentic, and free to express themselves when interacting with their child. *Note*: * *p* < .05, ** *p* < .01


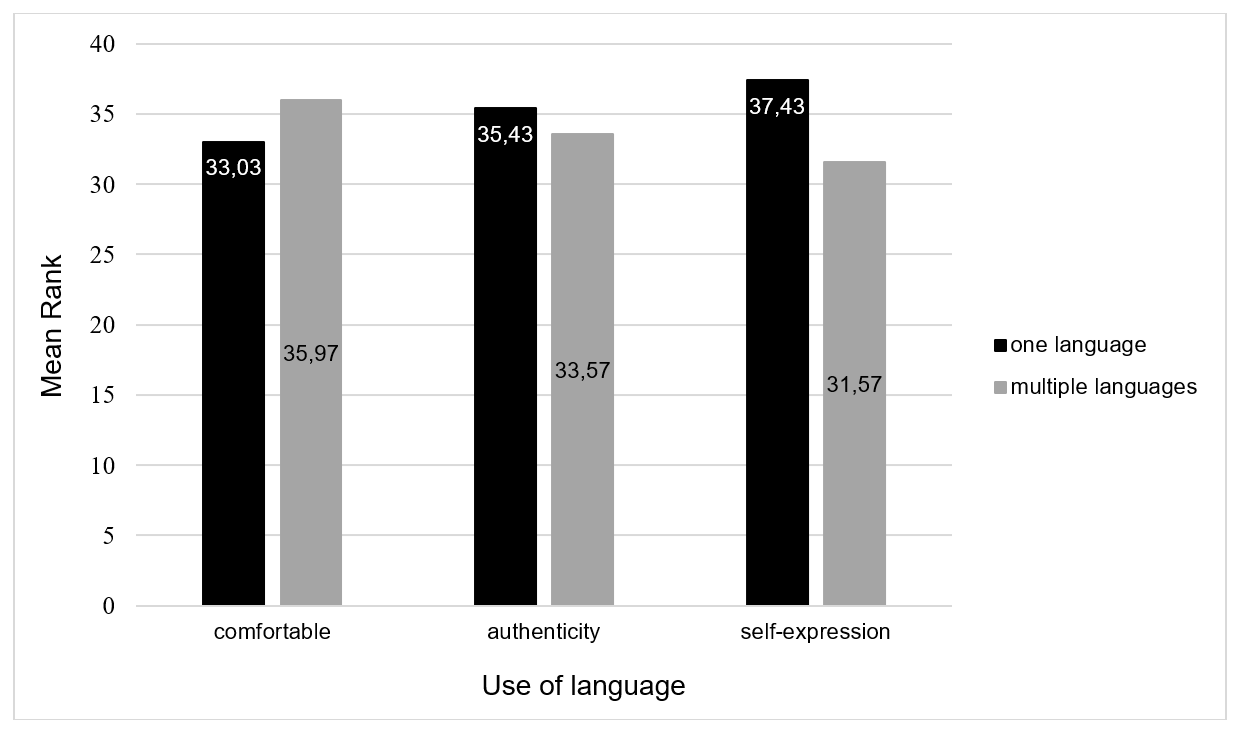


**Supplementary Figure 4***.* Mann-Whitney tests (mean ranks) comparing parents who used their mother tongue, either only their mother tongue or in combination with other languages, to parents who used either one or multiple foreign languages regarding feeling comfortable, authentic, and free to express themselves when interacting with their child. *Note*: * *p* < .05, ** *p* < .01


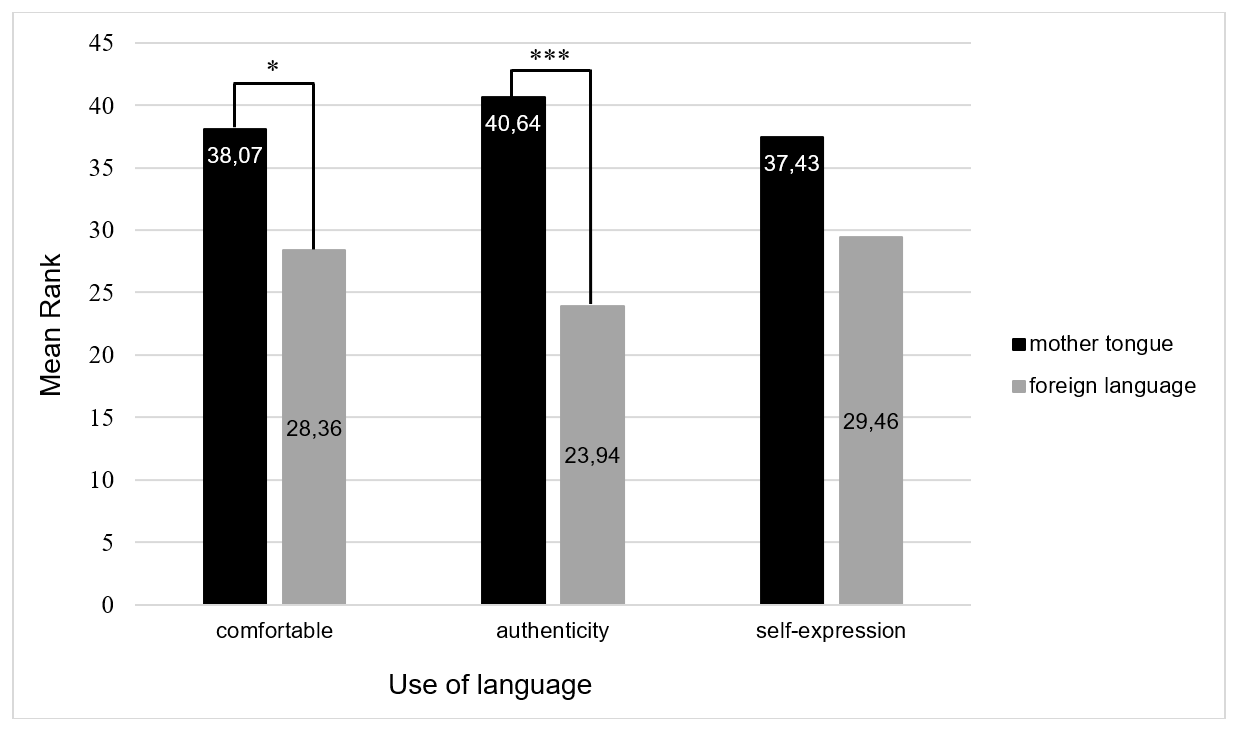

Supplement: Supplementary file 4 — Supplementary Material 4 [file 10803_2024_6347_MOESM4_ESM.docx]
